# Diffusion Tensor Imaging studies: white matter fibre alterations in Anorexia Nervosa (a systematic review)

**DOI:** 10.1186/2050-2974-3-S1-P11

**Published:** 2015-11-23

**Authors:** Beatriz Martin, Phillipa Hay, Stephen Touyz, Nasim Foroughi

**Affiliations:** 1 Western Sydney University, Sydney, Australia; 2 University of Sydney, Sydney, Australia

## Introduction

Diffusion Tensor Imaging technique (DTI) is a rapidly evolving procedure available for characterising abnormalities in white matter fibre structure (WM) in relation to neuropathology and treatment.

## Objective

to investigate the association of WM fibre tracts and microstructural alterations in Anorexia Nervosa (AN).

## Method

A systematic database search for published studies was conducted in November 2014 on 4 databases to identify studies that included samples of individuals with AN in which WM fibre tracts were analysed by means of DTI.

## Results

A total of 20 studies were retrieved, from which 6 met the inclusion criteria. The study samples were comprised of recovered patients (n=4), patients in the ill acute state (n=1), and both (n=1). Preliminary evidence suggests the WM fibre of the fornix, the cingulum, and the fronto-occipital fibre tracts are altered in AN patients but the persistence with or without recovery is less clear.

## Conclusion

The selected studies expose diverse altered tracts in AN, mainly related to the limbic system and some prefrontal areas. The varying findings may reflect its symptom complexity. The DTI technique appears to be well suited to examine the neurological underpinnings of AN. Further research is needed to study putative alterations in patients who recover and in those who do not recover.

